# Response of seedling growth and physiology of *Sorghum bicolor* (L.) Moench to saline-alkali stress

**DOI:** 10.1371/journal.pone.0220340

**Published:** 2019-07-30

**Authors:** Jingkuan Sun, Lei He, Tian Li

**Affiliations:** Shandong Key Laboratory of Eco-Environmental Science for Yellow River Delta, Binzhou University, Binzhou, P.R. China; Hainan University, CHINA

## Abstract

Soil salinization is a serious problem that affects the seedling growth in many regions. A greenhouse experiment was carried to investigate the adaptation ability of seedlings (*Sorghum bicolor* (L.) Moench.) in coastal saline alkaline environment. Seedlings of sorghum were treated by different salt and alkali stress (NaCl: Na_2_SO_4_: NaHCO_3_ were 2:1:0, 2:1:1, 2:1:2). The treatments consisted of three levels of salinity (100, 200 and 300 mmol/L) and pH values were 7.08, 8.78 and 9.04. The results showed that the seedlings of sorghum have good adaptability to salt stress under low pH (pH ≤7.08). The plant height, the maximum leave areas of seedlings all dropped and root length first ascended and then descended with the increasing of salt and alkali stress. The contents of Chlorophyll b degraded significantly under salt and alkali stress. Salt and alkali stress stimulated the accumulation of organic solutes (proline and protein) and inorganic ions (Na^+^, Cl^-^, SO_4_^2-^). Our results showed that salt and alkali stress have significant effect on growth indexes except root length and the interaction effect has significantly on physiology.

## Introduction

Soil salinization has become one of the most serious global environmental problems. There are more than 800 hectares salt-affected lands throughout the world [1]. Although a lot of considerable measures have been taken on this problem, Salt stress plays an important role in plant growth and physiological indexes [2–4]. When HCO_3_^–^ and /or CO_3_^2–^ are contained in saline soil, the soil pH (potential of hydrogen) increased, crops will be exposed to saline-alkali stress.

Seedling growth is the vitally important stage on the process of the plant growth. The adaptation of different seedlings to the environment may be different [5–6]. There are many reports on the physiological and ecological research of plants on salt stress, in generally, plant height, fresh and dry biomass, photosynthesis are inhibited by high levels of salt stress [7–10].

*Sorghum bicolor* is an energy plant with high biomass yield and a wild variety of ecological functions. It has good adaptability to salt stress and belongs to C_4_ plant with the high photosynthetic rate, which is considered to be one of the most potential energy plants [11]. It has great significance of exploring seedling growth mechanism under saline-alkaline stress for expanding the sorghum planting area, development and utilization of land resources and relieving the energy crisis.

## Materials and methods

### Plant material and cultivation condition

*Sorghum* Seeds were collected from institution of plant research in Chinese Academy of Sciences. Relatively uniform seeds were planted plastic pots(32 cm in diameter), filled with 8 kg of washed sand (through 2 mm sieve). After the seeds germination, 20 seedlings are contained in each pot, watered with Hoagland nutrient solution at 18:00 every day. All of the pots were placed in the green house with day average temperatures 22.5°Cand night average temperature 13.6°C.

### Stress treatment

According to the soil conditions in the Yellow River Delta, three salts (NaCl, Na_2_SO_4_, NaHCO_3_) mixed in an quality ratio were used for simulating saline-alkali stress. The treatment group labeled as A, B, C in order according to gradually increasing proportion of alkaline salts. The quality ratio of each group (NaCl:Na_2_SO_4_:NaHCO_3_ were 2:1:0, 2:1:1, 2:1:2. Treatments consisted of three levels of salinity (100, 200 and 300 mmol/L) in each of three pH levels: A (pH 7.08±0.02), B (pH 8.78±0.17), C (pH 9.04±0.14), the control groups were watered by Hoagland nutrient solution(CK). In each treatment group, with the increase of salt concentration, pH has no significant change because of buffer system. Stress treatments were according to the methods Shi and Li [2,10].

### Growth index determination

Two weeks later, the plants were harvested and washed clean. The roots, stems, and leaves of plants are separated, roots length, plants height, the maximum leaves areas were measured and weighed. Relative growth rate (RGR) was determined by the following formula[12].

$$\text{RGR}=\frac{\text{ln}\;\text{final}\;\text{dry}\;\text{biomass}-\text{ln}\;\text{initial}\;\text{dry}\;\text{biomass}}{\text{Duration}\;\text{of}\;\text{treatment}\;\left(\text{days}\right)}$$

### Physiological index determination

Physiological parameters were measured during the experimental period. Net photosynthetic rate were measured after one week treatment per cultivar per treatment combination using a CIRAS-2 portable photosynthesis system. The light intensity was maintained at 1200 μmol· m^-2^·s^-1^ on the third leaf.

Chlorophylls *a* and *b* and total concentrations were determined using compounding solution of 5 mL 80% acetone and 5 mL 95% ethanol. The sample was completely socked in the compounding solution in the test tube, and was measured until the color changing to white. Physiological parameters were measured during the experimental period. Superoxide dismutase (SOD), catalase (CAT), peroxidase (POD), malondialdehyde (MDA), the proline and total soluble proteins were measured by test kit from Jiancheng Bioengineering Institute, Nanjing, China.

### Inorganic cations and anions

Dry leave samples were homogenized by powdering, 400 mg dry leave samples were treated with acid digestion to measured the contents of K^+^, Na^+^, Ca^2+^ and Mg^2+^ by atomic absorption spectrophotometer (AA-6800). For Cl^-^, SO_4_^2-^, NO_3_^-^ determination, 100 mg of dry leave samples were taken and heated in 10 mL of distill water at 100°C, then determine the content by ion chromatography (ICS-2000).

### Statistical analysis

Data were analyzed by the SPSS 16.0 software package. Two-way ANOVA was performed to test the significance of main effects (salinity and pH) and their interaction on physiological indices.

## Results

### Response of seedling growth of sorghum to saline-alkali stress

**Fig 1:**
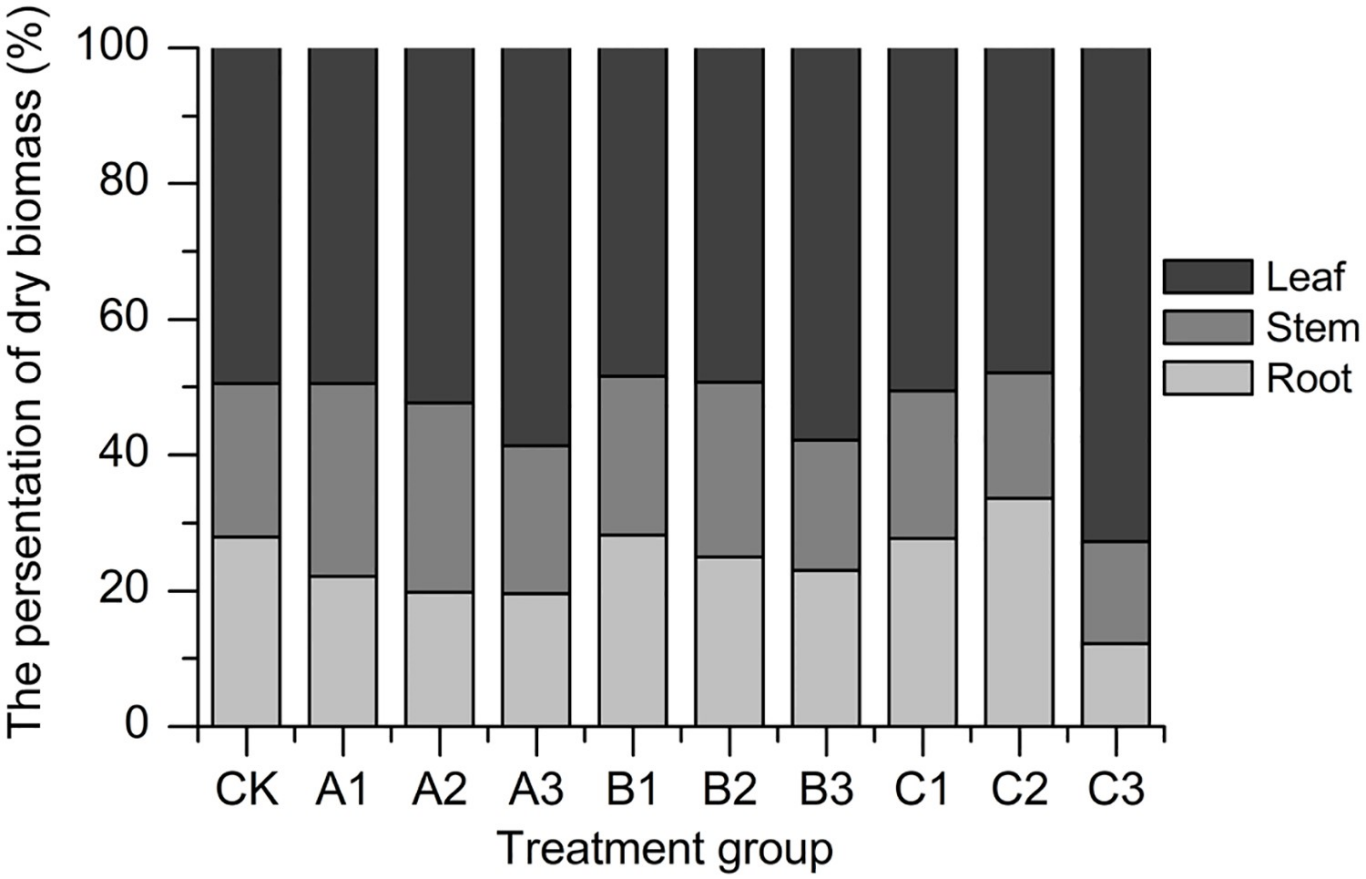
Response of the persentation of dry biomass of sorghum to saline-alkali stress. Note: Values represent means±S.E. Values at each treatment group followed by different letters are significantly different (P < 0.05).

Biomass decreased with increasing saline–alkali stress except for treatment group A1 and B2 (Table 1). With the increasing of salinity and alkalinity, the plant height and the maximum leave areas all dropped except for treatment group A1 (Table 1), which have the maximum values and were significant differences between the other groups. The greatest inhibition was the C_3_ treatment with the salinity was 300 mmol/L and the pH value was 9.04, plant height, the maximum leave areas, root length were respectively decreased to 43.66%, 78.47%, 29.17% comparing to the control. The biomass percentage of dry biomass of sorghum to saline-alkali stress was shown (Fig 1). The lowest biomass percentage of stem and leaf was found in C3.

**Table 1:** Plant height, the maximum leave areas, root length, biomass and relative growth rate (RGR) of *Sorghum bicolor* (L.) Moench seedlings under various salt and alkali stresses.

Treatment	Salinity	Plant height	Leaf area	Root length	Biomass	RGR(%)
	(mmol/)	(cm)	(cm^2^)	(cm)	(mg)	
CK	0	38.89±0.90a	46.03±1.41a	13.78±0.95a	374.10±21.75a	13.37±0.42a
A1	100	46.64±2.44b	60.16±7.54b	14.18±0.46a	379.20±19.17a	13.48±0.36a
A2	200	38.04±2.97ac	47.25±9.00ac	15.51±1.02ab	371.60±45.88a	13.25±0.84a
A3	300	31.45±1.09de	32.37±3.00d	14.74±0.37ac	246.40±11.82bc	10.40±0.35bd
B1	100	33.72±2.68ad	40.68±2.84ade	18.04±0.28b	340.60±11.71ad	12.72±0.25ac
B2	200	32.90±1.39cdf	34.10±2.90ae	16.99±1.22bce	406.20±7.75a	13.98±0.14a
B3	300	26.74±1.18egh	26.89±2.53f	13.28±0.97ad	215.30±19.68b	9.39±0.69b
C1	100	28.11±1.31fg	31.31±0.2672dg	14.97±0.44ade	293.30±41.75cd	11.52±0.98cd
C2	200	24.33±0.62gh	19.44±1.71h	15.00±0.54ade	204.60±4.16b	9.08±0.14b
C3	300	21.91±1.19h	9.91±1.46i	9.76±1.44f	128.30±4.50e	5.75±0.25e

Note: Values represent means±S.E. Values at each treatment group followed by different letters are significantly different (P < 0.05).

Growth index of sorghum seedlings were significantly affect by salinity (F_2,18_ = 20.206, *P*<0.001 for plant height; F_2,18_ = 17.044, *P*<0.001 for leaf area; F_2,18_ = 14.167, *P*<0.001 for root length; F_2,18_ = 33.545, *P*<0.001 for biomass; F_2,18_ = 52.137, *P*<0.001 for RGR) and alkalinity (F_2,18_ = 43.848, *P*<0.001 for plant height; F_2,18_ = 26.908, *P*<0.001 for leaf area; F_2,18_ = 8.572, *P*<0.01 for root length; F_2,18_ = 25.298, *P*<0.001 for biomass; F_2,18_ = 41.443, *P*<0.001 for RGR) (Table 2).

**Table 2:** Two-way ANOVA of effects of salinity (S), alkalinity (A), and their interactions on growth, physiological index organic solutes, inorganic cations and anions of *Sorghum bicolor* (L.) Moench seedlings.

	Source of variation					
	S		A		S×A	
Plant height/cm	20.206***	<0.001	43.848***	<0.001	2.280 n.s.	0.101
Leaf area/cm^2^	17.044***	<0.001	26.908***	<0.001	0.654 n.s.	0.632
Root length/cm	14.167***	<0.001	8.572**	<0.01	3.929*	<0.05
Biomass/mg	33.545***	<0.001	25.298***	<0.001	2.927n.s.	0.05
RGR (%)	52.137***	<0.001	41.443***	<0.001	3.809*	<0.05
Chl a /mg·g^-1^	5.482*	<0.05	16.867***	<0.001	8.304**	<0.01
Chl b /mg·g^-1^	5.661*	<0.05	3.285n.s.	0.061	1.171n.s.	0.356
Chl (a+b) /mg·g^-1^	6.790**	<0.01	12.742***	<0.001	5.813**	<0.01
Pn /umol·m^-2^·s^-1^	204.800***	<0.001	497.500***	<0.001	254.258***	<0.001
Proline content/mg ·g^-1^·DW	134.073***	<0.001	1.289n.s.	0.300	1.239n.s.	0.330
Soluble protein/ mg·g^-1^·FW	21.111***	<0.001	11.942**	<0.01	0.965n.s.	0.451
SOD activity/Umg^-1^prot	6.183**	<0.01	6.444**	<0.01	2.337n.s.	0.095
CAT activity /Umg^-1^prot·	1.447n.s.	0.261	4.174*	<0.05	1.389n.s.	0.277
POD activity /Umg^-1^prot·	10.749**	<0.01	4.307*	<0.05	3.912*	<0.05
MDA content /mmol·g^-1^prot	2.297n.s.	0.129	1.191n.s.	0.327	0.442n.s.	0.777
Na^+^ content	608.785***	<0.001	58.987***	<0.001	15.454***	<0.001
K^+^ content	9.205**	<0.01	97.962***	<0.001	11.382***	<0.001
Mg^2+^ content	17.130***	<0.001	7.815**	<0.01	0.380n.s.	0.820
Ca^2+^ content	22.355***	<0.001	12.882***	<0.001	6.844**	<0.01
Cl^-^ content	325.295***	<0.001	42.798***	<0.001	9.035***	<0.001
SO_4_^2-^content	216.531***	<0.001	110.247***	<0.001	10.853***	<0.001

Note: Data represent F-values at 0.05 level.

*p < 0.05;

**P < 0.01;

***P < 0.001;

n.s., non significant.

#### Response of chlorophyll and net photosynthetic rate of sorghum to saline-alkali stress

The content of chlorophyll a, chlorophyll (a+b) and net photosynthetic rate were significantly affected by the salinity (F_2,18_ = 5.482, *P*<0.05, F_2,18_ = 6.790, *P*<0.01, F_2,18_ = 204.800, *P*<0.001), alkalinity (F_2,18_ = 16.867, *P*<0.01, F_2,18_ = 12.742, *P*<0.001, F_2,18_ = 497.500, *P*<0.001) and the interaction of salinity and alkalinity (F_4,18_ = 8.304, *P*<0.01, F_4,18_ = 5.813, *P*<0.01, F_4,18_ = 254.258, *P*<0.001) (Table 2). Salinity and alkalinity reduce net photosynthetic rate, the maximum and minimum net photosynthetic rates were respectively in A1 and C3 treatment (Table 3).

**Table 3:** Chlorophyll a (Chl a), chlorophyll b (Chl b), chlorophyll (a+b) and net photosynthetic rate (Pn) of *Sorghum bicolor* (L.) Moench seedlings under various salt and alkali stresses.

Treatment	Salinity (mmol/L)	Chl a (mg·g^-1^)	Chl b (mg·g^-1^)	Chl (a+b) (mg·g^-1^)	Pn (μmol/m^2^/s)
CK	0	1.29±0.12ae	1.75±0.06a	3.04±0.08a	14.34±2.61a
A1	100	1.53±0.11ab	1.14±0.04bcd	2.67±0.15bcd	39.93±0.04b
A2	200	1.69±0.06bd	1.27±0.07b	2.96±0.13ac	35.30±0.25c
A3	300	1.38±0.04ace	1.12±0.01bcd	2.50±0.04bd	16.08±0.52a
B1	100	1.59±0.09bc	1.19±0.06bc	2.78±0.14abc	21.12±0.21d
B2	200	1.55±0.10ab	1.24±0.11bc	2.80±0.19abc	15.73±0.37a
B3	300	1.86±0.12d	1.22±0.03bc	3.07±0.14a	0.67±0.67e
C1	100	1.26±0.07e	1.09±0.03cd	2.35±0.10d	4.32±0.03f
C2	200	1.60±0.08bcd	1.24±0.01b	2.84±0.08abc	6.98±0.20g
C3	300	0.93±0.08f	0.98±0.04d	1.91±0.12e	0.12±0.04e

Note: Values represent means±S.E. Values at each treatment group followed by different letters are significantly different (P < 0.05)

#### Response of proline and soluble protein content of sorghum to saline-alkali stress

Proline content was significantly affected by salinity (F_2,18_ = 134.073, *P* < 0.001). Proteins content was significantly affected by salinity (F_2,18_ = 21.111, *P* < 0.001) and alkalinity (F_2,18_ = 11.942, *P* < 0.01) (Table 2).

**Fig 2:**
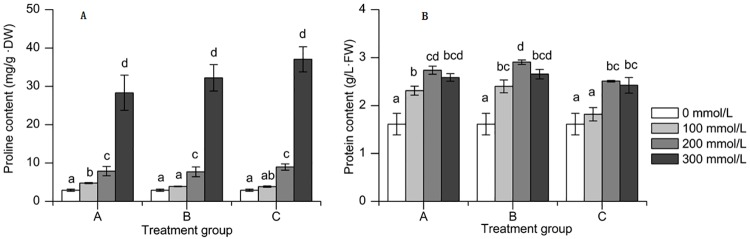
Response of proline and soluble protein content of sorghum to saline-alkali stress. (A)Proline content. (B) Soluble protein content. Note: Values represent means±S.E. Values at each treatment group followed by different letters are significantly different (P < 0.05).

Salinity stress stimulated the accumulation of proline (Fig 2A). In comparison with control, the proline contents of the leaves increased by 871.86%, 1005.16% and 1171.06% at A3, B3 and C3 treatment, respectively. The protein content in fresh leaves of sorghum seedlings showed a statistically significant between control plants and other treatment groups, and presented first ascended and then descended with the increasing of saline-alkali stress (Fig 2B). The maximum values in the A, B, C treatment group appeared in A2, B2, C2 treatment, the values were respectively 2.74 g/L, 2.90 g/L, 2.51 g/L, indicating that the high alkaline environment had a inhibition effect on synthesize of protein.

#### Response of antioxidative enzymes and MDA content of sorghum seedlings to saline-alkali stress

The protective enzyme system is sensitive to saline-alkali stress[13–14]. POD activity was significantly affected by the salinity (F_2,18_ = 10.749, *P* < 0.01), alkalinity (F_2,18_ = 4.307, *P* < 0.05) and the interaction of salinity and alkalinity (F_4,18_ = 3.912, *P* < 0.05). SOD activity was significantly affected by the salinity (F_2,18_ = 6.183, *P* < 0.01) and alkalinity (F_2,18_ = 6.444, *P* < 0.01) (Table 2). POD activity significantly increased with the increasing alkalinity under middle (200mmol/L) and high salt environment (300mmol/L). SOD activity significantly decreased under salt stress and in the low and middle salt environment, SOD activity was not significantly affected by the alkalinity (Table 4).

**Table 4:** Superoxide dismutase (SOD), catalase (CAT), peroxidase (POD), malondialdehyde (MDA) content of *Sorghum bicolor* (L.) Moench seedlings under various salt and alkali stresses.

Treatment	Salinity(mmol/L)	SOD(U/mg^-1^prot·)	CAT(U/mg^-1^prot·)	POD(U/mg^-1^prot·)	MDA(mmol·g^-1^prot)
CK	0	20.21±2.44a	7.91±0.97a	25.93±2.95a	3.69±0.37a
A1	100	15.31±0.04b	6.28±0.17ab	27.65±1.38a	5.10±0.34ab
A2	200	13.78±0.57bc	5.82±1.20abd	14.94±0.58b	5.91±1.07abd
A3	300	15.55±0.66b	4.37±0.11bc	14.63±2.46b	9.02±0.65bc
B1	100	14.50±0.87bc	4.77±0.44bc	23.76±4.21a	7.93±1.02ac
B2	200	11.25±0.44cd	3.40±0.74c	25.71±4.18a	6.24±0.81abd
B3	300	9.25±2.55d	3.28±0.65c	23.73±0.84ac	8.82±0.88bc
C1	100	15.70±0.99b	4.39±0.87bc	27.92±0.97a	6.79±0.99abd
C2	200	14.64±0.71bc	3.71±0.96cd	11.58±3.25b	9.25±2.07bc
C3	300	11.17±1.23cd	5.13±0.54bc	21.32±2.22bc	10.61±4.27cd

Note: Values represent means±S.E. Values at each treatment group followed by different letters are significantly different (P < 0.05)

#### Response of inorganic cations and anions of sorghum seedlings to saline-alkali stress

Inorganic cations (Na^+^ content, K^+^ content Mg^2+^ content and Ca^2+^ content) were significantly affected by salinity (F_2,18_ = 608.785, *P* < 0.001, F_2,18_ = 9.205, *P* < 0.01, F_2,18_ = 17.130, *P* < 0.001, F_2,18_ = 22.355, *P* < 0.001) and alkalinity (F_2,18_ = 58.987, *P* < 0.001, F_2,18_ = 97.962, *P* < 0.001, F_2,18_ = 7.815, *P* < 0.01, F_2,18_ = 12.882, *P* < 0.001) (Table 2).

**Fig 3:**
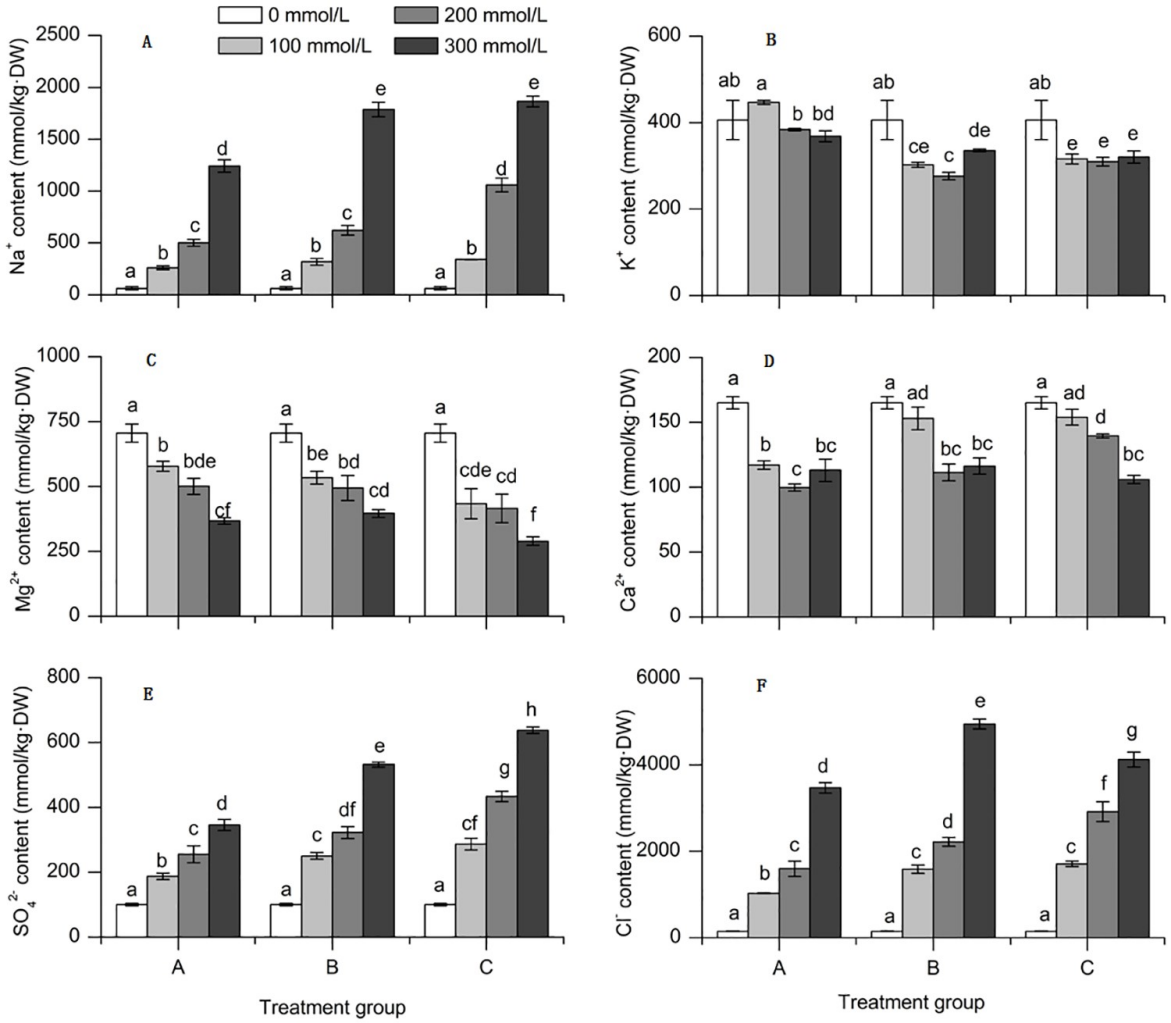
Response of inorganic cations and anions of sorghum seedlings to saline-alkali stress. (A)Na^+^ content. (B) K^+^ content. (C) Mg^2+^ content. (D) Ca^2+^ content. (E) SO_4_^2-^content. (F) Cl^-^content. Note: Values represent means±S.E. Values at each treatment group followed by different letters are significantly different (P < 0.05).

The Na^+^ and K^+^ of plants will be changed under saline-alkali stress[15]. Na^+^ content in leaves of sorghum sharply increased with the increasing of saline-alkali stress (Fig 3A). K^+^ content in leaves of sorghum decreased with the increasing of saline-alkali stress(Fig 3B). The Mg^2+^ content in leaves of sorghum seedlings were significant decreased(Fig 3C); the response to the Ca^2+^ accumulation in leaves were similar to the Mg^2+^(Fig 3D).

Inorganic anions (Cl^-^ content and SO_4_^2-^ content) were significantly affected by salinity (F_2,18_ = 325.295, *P* < 0.001, F_2,18_ = 216.531, *P* < 0.001), alkalinity (F_2,18_ = 42.798, *P* < 0.001, F_2,18_ = 110.247, *P* < 0.001) and the interaction of salinity and alkalinity (F_4,18_ = 9.035, *P* < 0.001, F_4,18_ = 10.853, *P* < 0.001) (Table 2). The two anions showed the same trend, with the increasing salinity and alkalinity, two anions content significant increased whereas Cl^-^ accumulated under various salt–alkali mixed stresses higher than SO_4_^2-^(Fig 3E and 3F). The maximum Cl^-^ and SO_4_^2-^ values appeared in B3 treatment group and C3 treatment group.

## Discussion

Seedling growth is a critical stage for the establishment of plant populations under saline-alkaline conditions [1]. The effect of different environmental conditions on seedling growth and physiology are different [7]. Generally, saline-alkali stress has serious effects on plant growth rate [2, 10], biomass, plant height, leaf areas and root length [16–17]. However, in the low salt and alkali environment, salinity and alkalinity stimulated the growth of sorghum seedlings [18–19]. Our studies found that the plant height, leaf areas, biomass in A1 treatment were greater than the controls (Table 1). The leaves biomass of sorghum seedlings accumulated with the increasing of saline-alkali stress, which account for 47.9%~72.7% of total dry weight. The percent of root biomass showed a downward trend because root was the first contacting organ to the treatment solution, which was demonstrated by the phenomenon that root accumulated more Na^+^ and suffered more serious iron toxicity [20].

In general, saline-alkali stress leads to a decrease in chlorophyll content and photosynthetic rates [3,10, 21–22]. In high alkali environment, chlorophyll content first increased and then decreased, indicating the moderate salt concentration can promote to synthesize more chlorophyll content, which can capture more light for photosynthesis use. Then in high salt-alkali environment (C3), the minimum chlorophyll content was 0.93mg/g, which showed that a high salt-alkali concentration could speed up the decomposition of chlorophyll content in the body of sorghum plants, reducing its photosynthetic efficiency [8, 23–24]. In this study, photosynthetic rate of sorghum increased under low saline environment., when the pH value was 9.04 and the salinity was 300 mmol/L (C3 treatment), the minimum net photosynthetic rates was 0.12 μmol·m^-2^·s^-1^, which is caused by non-stomatal factors [25].

In general, the osmotic regulators of plants contain inorganic ions and organic compounds [2, 26]. The compounds that accumulate most commonly are proline and soluble sugar, etc [27]. In this study, with the increasing of saline-alkali stress, proline content in leaves of sorghum seedlings increased in order to defense the stress. The protein content in fresh leaves of sorghum seedlings presented first ascended and then descended with the increasing of saline-alkali stress, which indicating that the high alkaline environment has a inhibition effect on synthesize of protein.

Under saline-alkali stress, plants will produce a large number of reactive oxygen products, resulting in damage to the plant membrane lipid peroxidation. The protective enzymes in plants play an important role in removing membrane lipid peroxidation [4, 22]. SOD is a key protective enzyme for the removal of reactive oxygen species, which can decompose reactive oxygen into H_2_O_2_ and O_2_, then CAT and POD turn H_2_O_2_ into H_2_O and O_2_ [28]. In this study, the SOD activity in fresh leaves of sorghum seedlings significantly decreased with the increasing salinity stress. The possible reason of this phenomenon is that saline-alkali stress blocks the pathway of enzyme synthesis and reduces enzyme activity [29]. In the low alkali environment, SOD activity was not affect by the salinity, which showed that SOD activity of sorghum seedlings has a certain adaptability to saline-alkali stress [30]. CAT activity showed the same trend of SOD. The content of MDA is an important sign of membrane lipid peroxidation damage in plants [25,31]. The MDA content increased with the increasing of saline-alkali stress, the maximum MDA value appeared in the C3 treatment, which showed that sorghum seedlings suffered severe oxidative damage under high salt- alkaline stress.

Meanwhile, plants usually accumulate inorganic ions in vacuoles under saline-alkali stress to reduce the cell water potential [32]. The environment of low Na^+^ and high K^+^ in the cytoplasm is a necessary condition to maintain the normal activity of a series of enzymes in plant cells. Saline-alkali stress can damage the balance of Na^+^ and K^+^ in the plant cytoplasm [33–35]. In this study, with the increasing of saline-alkali stress, Na^+^ content in leaves of sorghum seedlings increased (Fig 3). Conversely, K^+^ content decreased. This is because under saline-alkali stress, a large amount of Na^+^ enter the plant body, affecting the absorption of K^+^ and destroying the balance between Na^+^ and K^+^ in the plant [34].

In this study, with the increasing of saline-alkali stress, the Mg^2+^ content and Ca^2+^ in content leaves of sorghum seedlings were significant decreased. This shows that salt and alkali stress affects the absorption of Ca^2+^ and Mg^2+^ process [36]. The main effect on the plant leaf was to accelerate the plant senescence process with the Mg^2+^ deficiency [37]. Reduced the function of Ca^2+^ to maintain the integrity of the cell membrane and regulate the selective transport of Na^+^ and K^+^ [38].

In conclusion, sorghum seedlings were capable of surviving well under low pH (pH ≤ 7.08) regardless of the levels of salinity. Low and moderate salinity concentration can promote to synthesize more chlorophyll content and promote the photosynthetic rate. In order to adapt the saline environment, more proline and protein in the leaves of sorghum seedlings were accumulated with increasing salinity through osmotic adjustment. SOD activity was not changed regardless of the levels of salinity under low alkali environment. POD activity significantly increased with the increasing alkalinity under middle (200mmol/L) and high salt environment (300mmol/L). This results show that the seedlings of sorghum could adapt to salt- alkali stress environment by adjusting their physiological indexes.
